# Robust heavy-traffic approximations for service systems facing overdispersed demand

**DOI:** 10.1007/s11134-018-9584-z

**Published:** 2018-05-11

**Authors:** Britt W. J. Mathijsen, A. J. E. M. Janssen, Johan S. H. van Leeuwaarden, Bert Zwart

**Affiliations:** 1 Department of Mathematics and Computer Science, Eindhoven University of Technology, P.O. Box 513, 5600 MB Eindhoven, The Netherlands; 2 Centrum Wiskunde and Informatica, P.O. Box 94079, 1090 GB Amsterdam, The Netherlands

**Keywords:** Heavy-traffic approximations, Overdispersion, Saddle point method, Random walk, 60K25, 60G50, 30E20, 41A60

## Abstract

Arrival processes to service systems often display fluctuations that are larger than anticipated under the Poisson assumption, a phenomenon that is referred to as *overdispersion*. Motivated by this, we analyze a class of discrete-time stochastic models for which we derive heavy-traffic approximations that are scalable in the system size. Subsequently, we show how this leads to novel capacity sizing rules that acknowledge the presence of overdispersion. This, in turn, leads to robust approximations for performance characteristics of systems that are of moderate size and/or may not operate in heavy traffic.

## Introduction

One of the most prevalent assumptions in queueing theory is the assumption that the number of arrivals over any given period is a Poisson random variable with deterministic rate, whose variance equals its expectation. Although natural and convenient from a mathematical viewpoint, the Poisson assumption often fails to be confirmed in practice. Namely, a growing number of empirical studies show that the variance of demand typically deviates from the mean significantly. Recent work [24, 26] reports variance being strictly less than the mean in health care settings employing appointment booking systems. This reduction of variability can be accredited to the goal of the booking system to create a more predictable arrival pattern. On the other hand, in other scenarios with no control over the arrivals, the variance can dominate the mean; see [4–6, 11, 12, 17, 19, 23, 25, 30, 31, 34, 38, 41]. The feature that variability is higher than one expects from the Poisson assumption is referred to as *overdispersion* and serves as the primary motivation for this work.

Stochastic models with the Poisson assumption have been widely applied to optimize capacity levels in service systems. When stochastic models, however, do not take into account overdispersion, resulting performance estimates are likely to be overoptimistic. The system then ends up being underprovisioned, which possibly causes severe performance problems, particularly in critical loading.

A significant part of the queueing literature has focused on extending Poisson arrival processes to more bursty arrival processes, and analyzing these models using, for example, matrix-analytic models [29, 33]. In this paper, we focus on a different cause of overdispersion in arrival processes, which is *arrival rate uncertainty*. Since model primitives, in particular the arrival rate, are typically estimated through historical data, these are prone to be subject to forecasting errors. In the realm of Poisson processes, this inherent uncertainty can be acknowledged by viewing the arrival rate $\Lambda _n$ itself as being stochastic. The resulting doubly stochastic Poisson process, also known as a Cox process (first presented in [14]), implies that demand in a given interval $A_{k,n}$ follows a mixed Poisson distribution. In this case, the expected demand per period equals $\mu _n = {\mathbb {E}}[\Lambda _n]$, while the variance is $\sigma _n^2 = {\mathbb {E}}[\Lambda _n]+\mathrm{Var}\,\Lambda _n$. By selecting the distribution of the mixing factor $\Lambda _n$, the magnitude of overdispersion can be made arbitrarily large, and only a deterministic $\Lambda _n$ leads to a true Poisson process.

The mixed Poisson model presents a useful way to fit both the mean and variance to real data, particularly in case of overdispersion. The mixing distribution can be estimated parametrically or nonparametrically; see [23, 30]. A popular parametric family is the Gamma distribution, which gives rise to an effective data fitting procedure that uses the fact that a Gamma mixed Poisson random variable follows a negative binomial distribution. We will in this paper adopt the assumption of a Gamma–Poisson mixture as the demand process.

We investigate the impact of this modeling assumption within the context of a classical model in queueing theory, which is the reflected random walk. In particular, we consider a sequence of such random walks, indexed by *n*, with increments $A_{k,n}-s_n$, where $A_{k,n}\sim \,\mathrm{Pois}(\Lambda _n)$ and $s_n$ denotes the system capacity, and we consider a regime in which the system approaches heavy traffic. We are especially interested in the impact of overdispersion on the way performance measures scale, and how they impact capacity allocation rules.

A sensible candidate capacity allocation rule is $s_n = \mu _n + \beta \sigma _n + o(\sigma _n)$ for some $\beta >0$, which is asymptotically equivalent to the scaling

$$\begin{aligned} \frac{\mu _n}{\sigma _n}\,(1-\rho _n) \rightarrow \beta , \quad \text {for } n\rightarrow \infty , \end{aligned}$$

where $\rho _n := \mu _n/s_n$ denotes the utilization. We will verify mathematically that this is asymptotically the appropriate choice and our methods allow us to quantify the accuracy of the resulting performance formulae for finite systems. Studies that have addressed similar capacity allocation problems with stochastic arrival rates include [28, 30, 39, 40]. Of the aforementioned papers, our work best relates to [30], in the sense that we also assess the asymptotic performance of a queueing system having a stochastic arrival rate in heavy traffic. We therefore expand the paradigm of the quality-and-efficiency-driven (QED) regime, which relies on the popular square-root staffing rule $s_n = \mu _n + \beta \sqrt{\mu _n}$, in order to have it accommodated for overdispersed demand that follows from a doubly stochastic Poisson process.

The first part of our analysis relates to [37], in which a sequence of cyclically thinned queues, denoted by $G_n/G_n/1$ queues, is considered. Here, $G_n$ indicates that only every $n\mathrm{th}$ point of the original point process is considered. In this framework, it is shown that the stationary waiting time can be characterized as the maximum of a random walk, in which the increments grow indefinitely. Under appropriate heavy-traffic scaling, the authors prove convergence to a Gaussian random walk and moreover characterize the limits of the stationary waiting time moments. Our work differs with respect to [37] in the sense that we study a discrete-time model, rather than the continuous-time $G_n/G_n/1$ queue. Also, the presence of the overdispersion requires us to employ an alternative scaling.

Furthermore, our approach through Pollaczek’s formula allows us to derive estimates for performance measures in pre-limit, i.e., large but finite-size, systems. Mathematically, this second part of our analysis is related to previous work [22]. In particular, we use a refinement of the saddle point technique to establish our asymptotic estimates. The associated analysis is substantially more involved in the present situation, as we will explain in Sect. 4.

*Structure of the paper* The remainder of this paper is structured as follows. Our model is introduced in Sect. 2 together with some preliminary results. In Sect. 3, we derive the classical heavy-traffic scaling limits for the queue length process in the presence of overdispersed arrivals both for the moments and the distribution itself. Section 4 presents our main theoretical result, which provides a robust refinement to the heavy-traffic characterization of the queue length measures in pre-limit systems. In Sect. 5, we describe the numerical results and demonstrate the heavy-traffic approximation.

## Model description and preliminaries

We consider a sequence of discrete stochastic models, indexed by *n*, in which time is divided into periods of equal length. At the beginning of each period $k=1,2,3,\ldots$, new demand $A_{k,n}$ arrives to the system. The demands per period $A_{1,n},A_{2,n},\ldots$ are assumed independent and equal in distribution to some nonnegative integer-valued random variable $A_n$. For brevity, we define $\mu _n:= {\mathbb {E}}A_{n}$ and $\sigma _n^2 = \mathrm {Var}\,A_n$. The system has a service capacity $s_n\in {\mathbb {N}}$ per period, so we have the recursion

1 $$\begin{aligned} Q_{k+1,n} = \max \{Q_{k,n} + A_{k,n}-s_n,0\}, \quad k=0,1,2,\ldots , \end{aligned}$$

with $Q_{0,n} = 0$. The duality principle for random walks, see, for example [35, Sec. 7.1], shows that this expression is equivalent to

2 $$\begin{aligned} Q_{k+1,n} {\;\buildrel {d}\over = \;}\max _{0\le j\le k}\left\{ {\sum _{i=1}^j} (A_{i,n}-s_n)\right\} , \quad k=0,1,2,\ldots , \end{aligned}$$

i.e., the maximum of the first *k* steps of a random walk with steps distributed as $A_n-s_n$. Even more, we can characterize $Q_{n}$, the stationary queue length, as

3 $$\begin{aligned} Q_{n} {\;\buildrel {d}\over = \;}\max _{k\ge 0}\left\{ {\sum _{i=1}^k} (A_{i,n}-s_n)\right\} . \end{aligned}$$

The behavior of $Q_{k,n}$ greatly depends on the characteristics of $A_n$ and $s_n$. First, note that $\mu _n<s_n$ is a necessary condition for the maximum to be finite and therefore for the queue to be stable. This random variable is finite a.s. if ${\mathbb {E}}[A_{i,n}]< s_n$, which is guaranteed by our Assumption 1 (in particular $\beta >0$) below. Before continuing the analysis of $Q_{n}$, we impose a set of conditions on the asymptotic properties of $s_n,\mu _n$ and $\sigma _n$, which are assumed to hold throughout the remainder of this paper.

### Assumption 1

- (Asymptotic growth)
  $$\begin{aligned} \mu _n,\sigma _n \rightarrow \infty , \quad \text { for } n\rightarrow \infty . \end{aligned}$$
- (Persistence of overdispersion)
  $$\begin{aligned} \sigma _n^2/\mu _n \rightarrow \infty , \quad \text { for } n\rightarrow \infty . \end{aligned}$$
- (Heavy-traffic condition) The utilization $\rho _n := \mu _n/s_n \rightarrow 1$ as $n\rightarrow \infty$ according to
  4 $$\begin{aligned} (1-\rho _n)\frac{\mu _n}{\sigma _n} \rightarrow \beta , \quad \text {for } n\rightarrow \infty , \end{aligned}$$
  for some $$\beta > 0$$.

By Assumption 1(a), we insist that the expected demand per period grows infinitely large, which allows us to develop approximations for systems with large yet finite arrival volumes. Moreover, it is assumed that the order of stochastic variability of the arrival process relative to the mean arrival volume does not vanish in the limit. In fact, the assumption on the persistence of overdispersion says that the variance of the demand per period is of higher order than its mean as *n* grows large. We note that the scenario with $\sigma _n^2/\mu _n\rightarrow \gamma$ for some $\gamma > 0$ is asymptotically equivalent to the process studied in [22], in which case overdispersion of the arrival process does not play a role in the limit as $n\rightarrow \infty$. In order to establish heavy-traffic approximations for large systems that do face overdispersion we need to construct an asymptotic regime in which overdispersion continues to play a dominant role as $n\rightarrow \infty$, which is secured by Assumption 1(b). The subsequent analysis will clarify why the heavy-traffic condition in Assumption 1(c) is the correct one for our purposes. Note that Assumption 1(c) is satisfied for the capacity allocation rule

5 $$\begin{aligned} s_n = \mu _n + \beta \, \sigma _n. \end{aligned}$$

Since we are mainly interested in the system behavior in heavy traffic, it is appropriate to study the queue length process in a scaled form. Substituting $s_n$ as in Assumption 1(c), and dividing both sides of (3) by $\sigma _n$, gives

6 $$\begin{aligned} \frac{Q_{n}}{\sigma _n} = \max _{k\ge 0} \left\{ {\sum _{i=1}^k} \left( \frac{A_{i,n}-\mu _n}{\sigma _n} - \beta \right) \right\} . \end{aligned}$$

By defining ${\hat{Q}}_n := Q_n/\sigma _n$ and

7 $$\begin{aligned} {\hat{A}}_{i,n} := (A_{i,n}-\mu _n)/\sigma _n, \end{aligned}$$

we see that the scaled queue length process is in distribution equal to the maximum of a random walk with i.i.d. increments distributed as ${\hat{A}}_n-\beta$. Besides ${\mathbb {E}}{\hat{A}}_n = 0$ and $\mathrm{Var}\,{\hat{A}}_n=1$, the scaled and centered arrival counts ${\hat{A}}_n$ have a few other nice properties which we turn to later in this section.

The model in (1) is valid for any distribution of $A_n$, also for the original case where the number of arrivals follows a Poisson distribution with fixed parameter $\lambda _n$, but Assumption 1(b) does not hold then. Instead, we assume $A_n$ to be Poisson distributed with uncertain arrival rate rendered by the nonnegative random variable $\Lambda _n$. This $\Lambda _n$ is commonly referred to as the *prior* distribution, while $A_n$ is given the name of a Poisson mixture; see [18]. Given that the moment generation function of $\Lambda _n$, denoted by $M^\Lambda _n(\cdot )$, exists, we are able to express the probability generating function (pgf) of $A_n$ through the former. Namely,

8 $$\begin{aligned} {\tilde{A}}_n(z) = {\mathbb {E}}[{\mathbb {E}}[ z^{A_n} | \Lambda _n ] ] = {\mathbb {E}}[ \exp (\Lambda _n(z-1))] = M^\Lambda _n(z-1). \end{aligned}$$

From (8), we get

9 $$\begin{aligned} \mu _n = {\mathbb {E}}A_n = {\mathbb {E}}\Lambda _n,\quad \sigma _n^2 = \mathrm{Var}\,A_n = \mathrm{Var}\,\Lambda _n + {\mathbb {E}}\Lambda _n, \end{aligned}$$

so that $\mu _n<\sigma _n^2$ if $\Lambda _n$ is non-deterministic. Assumption 1(b) hence translates to

$$\begin{aligned} \mathrm{Var}\,\Lambda _n/{\mathbb {E}}\Lambda _n\rightarrow \infty , \quad n\rightarrow \infty . \end{aligned}$$

The next result relates the convergence behavior of the centered and scaled $\Lambda _n$ to that of ${\hat{A}}_n$.

### Lemma 1

Let $\mu _n,\sigma _n^2\rightarrow \infty$ and $\sigma _n^2/\mu _n\rightarrow \infty$. If

10 $$\begin{aligned} {\hat{\Lambda }}_n := \frac{\Lambda _n-\mu _n}{\sigma _n}{\;\buildrel {d}\over \Rightarrow \;}N(0,1), \quad \text { for } n\rightarrow \infty , \end{aligned}$$

where *N*(0, 1) denotes a standard normal variable, then ${\hat{A}}_n$ converges weakly to a standard normal variable as $n\rightarrow \infty$.

The proof can be found in Appendix A.

The prevalent choice for $\Lambda _n$ is the Gamma distribution. The Gamma–Poisson mixture turns out to provide a good fit to arrival counts observed in service systems, as was observed by [23, 30]. Assuming $\Lambda _n$ to be of Gamma type with scale and rate parameters $a_n$ and $1/b_n$, respectively, we get

11 $$\begin{aligned} {\tilde{A}}_n(z) = \left( \frac{1}{1+b_n(1-z)}\right) ^{a_n}, \end{aligned}$$

in which we recognize the pgf of a negative binomial distribution with parameters $a_n$ and $1/(b_n+1)$, so that

12 $$\begin{aligned} \mu _n = a_nb_n,\quad \sigma _n^2 = a_nb_n(b_n+1). \end{aligned}$$

Note that in the context of a Gamma prior, the restrictions in Assumption 1 reduce to only two rules. For completeness, we include the revised list below.

### Assumption 2

- (Asymptotic regime and persistence of overdispersion)
  $$\begin{aligned} a_n, b_n \rightarrow \infty , \quad \text { for } n\rightarrow \infty . \end{aligned}$$
- (Heavy-traffic condition) Let
  $$\begin{aligned} s_n = a_n b_n + \beta \sqrt{a_n b_n(b_n+1)} + o\big ( \sqrt{a_n} b_n \big ), \end{aligned}$$
  for some $\beta >0$, or equivalently
  $$\begin{aligned} (1-\rho _n)\sqrt{a_n} \rightarrow \beta , \quad \text { for } n\rightarrow \infty . \end{aligned}$$

The next result follows from the fact that $\Lambda _n$ is a Gamma random variable:

### Corollary 1

Let $\Lambda _n\sim \text { Gamma}(a_n,1/b_n)$, $A_n\sim \mathrm{Poisson }(\Lambda _n)$ and $a_n,b_n\rightarrow \infty$. Then, ${\hat{A}}_n$ converges weakly to a standard normal random variable as $n\rightarrow \infty$.

### Proof

With Lemma 1 in mind, it is sufficient to prove that ${\hat{\Lambda }}_n\Rightarrow N(0,1)$ for this particular choice of $\Lambda _n$. We do this by proving the pointwise convergence of the characteristic function (cf) of ${\hat{\Lambda }}_n$ to $\exp ({-} t^2/2)$, the cf of the standard normal distribution. Let $\varphi _{G}(\cdot )$ denote the characteristic function of a random variable *G*. By basic properties of the cf,

13 $$\begin{aligned} \varphi _{{\hat{\Lambda }}_n}(t)&= \mathrm{e}^{-i\mu _nt/\sigma _n}\,\varphi _{\Lambda _n}(t/\sigma _n) = \mathrm{e}^{-i\mu _nt/\sigma _n} \left( 1-\frac{i b_nt}{\sigma _n}\right) ^{-a_n}\nonumber \\&= \exp \left[ -\frac{i\mu _nt}{\sigma _n}\, - a_n\,\mathrm{ln}\left( 1-\frac{i b_nt}{\sigma _n}\right) \right] \nonumber \\&= \exp \left[ -\frac{i\mu _nt}{\sigma _n} -a_n\left( {-}\frac{i\,b_nt}{\sigma _n} + \frac{b_n^2t^2}{2\sigma _n^2} + O( b_n^3/\sigma _n^3)\right) \right] \nonumber \\&= \exp \left[ -\frac{b_n\,t^2}{2(b_n+1)} + O\left( 1/\sqrt{a_n}\right) \right] \rightarrow \exp \left( {-} t^2/2\right) , \end{aligned}$$

for $n\rightarrow \infty$. Since $b_n/\sigma _n = a_n^{-1/2}(1+1/b_n)^{-1/2}\rightarrow 0$ as $n\rightarrow \infty$, we can take the principal value in (13) for the logarithm when *t* is in any compact set and *n* is large enough. By Lévy’s continuity theorem, see, for example, [16, Thm. 18.21], this implies ${\hat{\Lambda }}_n$ is indeed asymptotically standard normal. $\square$

The characterization of the arrival process as a Gamma–Poisson mixture is of vital importance in later sections.

### Expressions for the stationary distribution

Our main focus is on the stationary queue length distribution, denoted by

$$\begin{aligned} {\mathbb {P}}(Q_{n}=i) =\lim _{k\rightarrow \infty } {\mathbb {P}}(Q_{k,n}=i). \end{aligned}$$

Denote the pgf of $Q_{n}$ by

14 $$\begin{aligned} {\tilde{Q}}_n(w) = \sum _{i=0}^\infty {\mathbb {P}}(Q_{n}=i) w^i. \end{aligned}$$

To continue our analysis of $Q_{n}$, we need one more condition on $A_n$.

#### Assumption 3

The pgf of $A_n$, denoted by ${\tilde{A}}_n(w)$, exists within $|w|<r_0$, for some $r_0>1$, so that all moments of $A_n$ are finite.

We next recall two characterizations of ${\tilde{Q}}_n(w)$ that play prominent roles in the remainder of our analysis. The first characterization of ${\tilde{Q}}_n(w)$ originates from a random walk perspective. As we see from (3), the (scaled) stationary queue length is equal in distribution to the all-time maximum of a random walk with i.i.d. increments distributed as $A_n-s_n$ (or ${\hat{A}}_n-\beta$ in the scaled setting). Spitzer’s identity, see, for example, [3, Theorem VIII4.2], then gives

15 $$\begin{aligned} {\tilde{Q}}_n(w) = \exp \left\{ \sum _{k=1}^\infty \frac{1}{k}\,\left( {\mathbb {E}}\left[ w^{\left( \sum _{i=1}^k \{A_{i,n}-s_n\}\right) ^+}\right] -1\right) \right\} , \end{aligned}$$

where $$(x)^+ = \max \{x,0\}$$. Hence,

16 $$\begin{aligned} {\mathbb {E}}Q_{n}= & {} {\tilde{Q}}_n'(1) = \sum _{k=1}^\infty \frac{1}{k}{\mathbb {E}}\left[ {\sum _{i=1}^k} (A_{i,n} - s_n) \right] ^+, \end{aligned}$$

17 $$\begin{aligned} \mathrm{Var}\,Q_{n}= & {} {\tilde{Q}}_n''(1)+Q_n'(1)-\left( {\tilde{Q}}_n'(1)\right) ^2 = \sum _{k=1}^\infty \frac{1}{k}{\mathbb {E}}\left[ \left( {\sum _{i=1}^k} (A_{i,n} - s_n) \right) ^+\right] ^2, \end{aligned}$$

18 $$\begin{aligned} {\mathbb {P}}(Q_{n}=0)= & {} {\tilde{Q}}_n(0) = \exp \left\{ {-}{\sum _{k=1}^\infty }\frac{1}{k} {\mathbb {P}}\left( \sum _{i=1}^k (A_{i,n}-s_n) > 0\right) \right\} . \end{aligned}$$

A second characterization follows from Pollaczek’s formula, see [1, 22]:

19 $$\begin{aligned} {\tilde{Q}}_n(w) = \exp \left\{ \frac{1}{2\pi i}\int _{|z|=1+\varepsilon } \mathrm{ln}\left( \frac{w-z}{1-z}\right) \,\frac{(z^{s_n}-{\tilde{A}}_n(z))'}{z^{s_n}-{\tilde{A}}_n(z)}\, dz\right\} , \end{aligned}$$

which is analytic for $|w|<r_0$, for some $r_0>1$. Therefore, $\varepsilon >0$ has to be chosen such that $|w|<1+\varepsilon <r_0$. This gives

20 $$\begin{aligned} {\mathbb {E}}Q_{n}= & {} \frac{1}{2\pi i} \int _{|z|=1+\varepsilon } \frac{1}{1-z}\,\frac{(z^{s_n}-{\tilde{A}}_n(z))'}{z^{s_n}-{\tilde{A}}_n(z)}\, \mathrm{d}z, \end{aligned}$$

21 $$\begin{aligned} \mathrm{Var}\,Q_{n}= & {} \frac{1}{2\pi i} \int _{|z|=1+\varepsilon } \frac{{-}z}{(1-z)^2}\,\frac{(z^{s_n}-{\tilde{A}}_n(z))'}{z^{s_n}-{\tilde{A}}_n(z)}\, \mathrm{d}z, \end{aligned}$$

22 $$\begin{aligned} {\mathbb {P}}(Q_{n}=0)= & {} \exp \left\{ \frac{1}{2\pi i}\int _{|z|=1+\varepsilon } \mathrm{ln}\left( \frac{z}{z-1}\right) \,\frac{(z^{s_n}-{\tilde{A}}_n(z))'}{z^{s_n}-{\tilde{A}}_n(z)}\, \mathrm{d}z\right\} . \end{aligned}$$

Pollaczek-type integrals like (19)–(22) first occurred in the work of Pollaczek on the classical single-server queue (see [1, 13, 21] for historical accounts). These integrals are fairly straightforward to evaluate numerically and hence give rise to efficient algorithms for performance evaluation [1, 9]. The integrals also proved useful in establishing heavy-traffic results by asymptotic evaluation of the integrals in various heavy-traffic regimes [8, 13, 22, 27], and in this paper we follow that approach for a heavy-traffic regime that is suitable for overdispersion.

## Heavy-traffic limits

In this section, we present the result on the convergence of the discrete process ${\hat{Q}}_{n}$ to a non-degenerate limiting process and of the associated stationary moments. The latter requires an interchange of limits. Using this asymptotic result, we derive two sets of approximations for ${\mathbb {E}}Q_n$, $\mathrm{Var}\,Q_n$ and ${\mathbb {P}}(Q_{n}=0)$ that capture the limiting behavior of $Q_{n}$. The first set provides a rather crude estimation for the first cumulants of the queue length process for any arrival process $A_{n}$ satisfying Assumption 1. The second set, which is the subject of the next section, is derived for the specific case of a Gamma prior and is therefore expected to provide more accurate, robust approximations for the performance metrics.

We start by indicating how the asymptotic properties of the scaled arrival process give rise to a proper limiting random variable describing the stationary queue length. The asymptotic normality of ${\hat{A}}_{n}$ provides a link with the Gaussian random walk and nearly deterministic queues [36, 37]. The main results in [36, 37] were obtained under the assumption that $\rho _n\sim 1-\beta /\sqrt{n}$, in which case it follows from [37, Thm. 3] that the rescaled stationary waiting time process converges to a reflected Gaussian random walk.

We shall also identify the Gaussian random walk as the appropriate scaling limit for our stationary system. However, since the normalized natural fluctuations of our system are given by $\mu _n/\sigma _n$ instead of $\sqrt{n}$, we assume that the load grows like $\rho _n \sim 1 - \frac{\beta }{\mu _n/\sigma _n}$. Hence, in contrast to [36, 37], our systems’ characteristics display larger natural fluctuations, due to the mixing factor that drives the arrival process. Yet, by matching this overdispersed demand with the appropriate hedge against variability, we again obtain Gaussian limiting behavior. This is not surprising, since we saw in Lemma 1 that the increments start resembling Gaussian behavior for $n\rightarrow \infty$. The following result summarizes this.

### Theorem 1

Let $A_n$ be a nonnegative random variable such that ${\hat{A}}_n = (A_n-\mu _n)/\sigma _n$ is asymptotically standard normal, with $\mu _n$ and $\sigma _n$ as defined in (9), and ${\mathbb {E}}[(\max \{{\hat{A}}_n,0\})^4]$ is bounded in *n*. Then, under Assumption 1, for $n\rightarrow \infty$,

(i) $${\hat{Q}}_{n} {\;\buildrel {d}\over \Rightarrow \;}M_\beta $$,
(ii) $${\mathbb {P}}(Q_{n} = 0) \rightarrow {\mathbb {P}}(M_\beta =0)$$,
(iii) $${\mathbb {E}}{\hat{Q}}_{n} \rightarrow {\mathbb {E}}M_\beta $$,
(iv) $$\mathrm{Var}\,{{\hat{Q}}}_n \rightarrow \mathrm{Var}\,\, M_\beta $$,

where $M_\beta$ is the all-time maximum of a random walk with i.i.d. normal increments with mean $-\beta$ and unit variance.

The proof of Theorem 1 is given in Appendix A. We remark that for convergence of the mean scaled queue length, only ${\mathbb {E}}[(\max \{{\hat{A}}_n,0\})^3]<\infty$ is needed. The following result shows that Theorem 1 also applies to Gamma mixtures, which is a direct consequence of Corollary 1.

### Corollary 2

Let $\Lambda _n\sim$ Gamma$(a_n,b_n)$. Then, under Assumption 2 the four convergence results of Theorem 1 hold true.

It follows from Theorem 1 that the scaled stationary queueing process converges under (4) to a reflected Gaussian random walk. Hence, the performance measures of the original system should be well approximated by the performance measures of the reflected Gaussian random walk, yielding heavy-traffic approximations.

Like our original system, the Gaussian random walk falls in the classical setting of the reflected one-dimensional random walk, whose behavior is characterized by both Spitzer’s identity and Pollaczek’s formula. In particular, Pollaczek’s formula gives rise to contour integral expressions for performance measures that are easy to evaluate numerically, also in heavy-traffic conditions. The numerical evaluation of such integrals is considered in [1]. For ${\mathbb {E}}M_\beta$, such an integral is as follows:

23 $$\begin{aligned} {\mathbb {E}}M_\beta = {-}\frac{1}{\pi }\int _0^\infty \mathrm{Re}\left[ \frac{1-\phi (-z)}{z^2}\right] \mathrm{d}y, \end{aligned}$$

with $\phi (z) = \exp (-\beta \,z+\tfrac{1}{2}\,z^2)$, the Laplace transform of a normal random variable with mean $-\beta$ and unit variance, and $z=x+iy$ with an appropriately chosen real part *x*. Note that this integral involves complex-valued functions with complex arguments. Similar Pollaczek-type integrals exist for ${\mathbb {P}}(M_\beta =0)$ and $\mathrm{Var}\,M_\beta$; see [1]. The following result simply rewrites these integrals in terms of real integrals and uses the fact that the scaled queue length process mimics the maximum of the Gaussian random walk for large *n*.

### Corollary 3

Under Assumption 1, the leading order behavior of ${\mathbb {P}}(Q_{n}=0)$, ${\mathbb {E}}Q_n$ and $\mathrm{Var}\,Q_n$ as $n\rightarrow \infty$ is given by, respectively,

24 $$\begin{aligned}&\exp \left[ \frac{1}{\pi } \int _0^\infty \frac{\beta /\sqrt{2}}{\tfrac{1}{2}\beta ^2+t^2}\,\mathrm{ln}\left( 1-\hbox {e}^{-\tfrac{1}{2}\beta ^2-t^2}\right) \mathrm{d}t\right] , \end{aligned}$$

25 $$\begin{aligned}&\frac{\sqrt{2}\sigma _n}{\pi }\int _0^\infty \frac{t^2}{\tfrac{1}{2}\beta ^2+t^2}\, \frac{\exp (-\tfrac{1}{2}\beta ^2- t^2)}{1-\exp (-\tfrac{1}{2} \beta ^2 - t^2)} \mathrm{d}t, \end{aligned}$$

26 $$\begin{aligned}&\frac{\sqrt{2}\beta \sigma _n^2}{\pi }\,\int _0^\infty \frac{t^2}{(\tfrac{1}{2} \beta ^2+t^2)^2}\frac{\exp (-\tfrac{1}{2}\beta ^2- t^2)}{1-\exp (-\tfrac{1}{2} \beta ^2 - t^2)} \mathrm{d}t. \end{aligned}$$

### Proof

According to [1, Eq. (15)],

$$\begin{aligned} {-}\,\mathrm{ln}\,[{\mathbb {P}}(M_\beta =0)] = c_0,\quad {\mathbb {E}}M_\beta = c_1, \quad \mathrm{Var}\,\, M_\beta = c_2, \end{aligned}$$

where

$$\begin{aligned} c_n = \frac{(-1)^nn!}{\pi } \,\mathrm{Re}\left[ \int _0^\infty \frac{\mathrm{ln}\,(1-\exp (\beta \,z+\tfrac{1}{2} z^2))}{z^{n+1}} \mathrm{d}y\right] , \end{aligned}$$

in which $z={-}x+i\,y$, $y\ge 0$, and *x* is any fixed number between 0 and $2\beta$. Take $x=\beta$, so that

$$\begin{aligned} \beta z+\tfrac{1}{2} z^2 = {-}\tfrac{1}{2}\beta ^2 - \tfrac{1}{2} y^2\le 0,\quad y\ge 0. \end{aligned}$$

For $n=0$, this gives

$$\begin{aligned} c_0&= \frac{1}{\pi }\,\mathrm{Re}\left[ \int _0^\infty \frac{\mathrm{ln}\,(1-\exp ({-}\tfrac{1}{2} \beta ^2-\tfrac{1}{2} y^2))}{{-}\beta +i\,y} \mathrm{d}y\right] \\&= {-}\frac{1}{\pi }\,\int _0^\infty \frac{\beta }{\beta ^2+y^2}\,\mathrm{ln}\,\left( 1-\exp ({-}\tfrac{1}{2} \beta ^2- \tfrac{1}{2} y^2)\right) \mathrm{d}y\\&= {-}\frac{1}{\pi }\,\int _0^\infty \frac{\beta /\sqrt{2}}{\tfrac{1}{2}\beta ^2+t^2}\,\mathrm{ln}\,\left( 1-\exp ({-}\tfrac{1}{2} \beta ^2-t^2)\right) \mathrm{d}t, \end{aligned}$$

where we used that

$$\begin{aligned} \mathrm{Re }\left[ \frac{1}{{-}\beta +i\, y}\right] = \frac{{-}\beta }{\beta ^2+y^2}, \end{aligned}$$

together with the substitution $y=t\sqrt{2}$. For $n=1,2,\ldots ,$ partial integration gives

$$\begin{aligned} c_n&= \frac{(-1)^n n!}{\pi } \, \mathrm{Re}\left[ \int _0^\infty \frac{\mathrm{ln}(1-\exp (-\tfrac{1}{2}\beta ^2-\tfrac{1}{2} y^2))}{({-}\beta +i\,y)^{n+1}} \mathrm{d}y\right] \\&= \frac{(-1)^{n-1}(n-1)!}{\pi }\,\mathrm{Im}\left[ \int _0^\infty \mathrm{ln}(1-\exp (-\tfrac{1}{2}\beta ^2-\tfrac{1}{2} y^2))\mathrm{d}\left( \frac{1}{(-\beta +i\,y)^n}\right) \right] \\&= {-}\frac{(-1)^{n-1}(n-1)!}{\pi } \mathrm{Im}\left[ \int _0^\infty \frac{y}{(-\beta +i\,y)^n}\,\frac{\exp (-\tfrac{1}{2}\beta ^2-\tfrac{1}{2} y^2)}{1-\exp (-\tfrac{1}{2}\beta ^2-\tfrac{1}{2} y^2)}\mathrm{d}y\right] , \end{aligned}$$

where we have used that

$$\begin{aligned} \mathrm{Im}\left[ \frac{\mathrm{ln}(1-\exp (-\tfrac{1}{2}\beta ^2-\tfrac{1}{2} y^2))}{(-\beta +i\,y)^n}\right] \Bigl |_0^\infty \Bigr . = 0. \end{aligned}$$

Using

$$\begin{aligned} \frac{1}{(-\beta +i\,y)^n} = (-1)^n\,\frac{(\beta +i\,y)^n}{(\beta ^2+y^2)^n}, \end{aligned}$$

we then get

$$\begin{aligned} c_n = \frac{(n-1)!}{\pi }\,\mathrm{Im}\,\left[ \int _0^\infty \frac{y(\beta +i\,y)^n}{(\beta ^2+y^2)^n}\,\frac{\exp (-\tfrac{1}{2}\beta ^2-\tfrac{1}{2} y^2)}{1-\exp (-\tfrac{1}{2}\beta ^2-\tfrac{1}{2} y^2)}\mathrm{d}y\right] , \end{aligned}$$

which, after substitution of $y=t\sqrt{2}$ gives (25) and (26). $\square$

## Robust heavy-traffic approximations

We shall now establish robust heavy-traffic approximations for the canonical case of Gamma–Poisson mixtures; see (11).

### Theorem 2

Let $a_n,b_n$ and $s_n$ be as in Assumption 2. Then, the leading order behavior of ${\mathbb {E}}Q_n$ is given by

27 $$\begin{aligned} \frac{\sqrt{2}\,\beta _n}{\pi }\left( \frac{b_n+\rho _n}{1-\rho _n}\right) \,\int _{0}^\infty \frac{t^2}{\tfrac{1}{2}\beta ^2_n+t^2}\,\frac{\exp ({-}\tfrac{1}{2}\beta ^2_n-t^2)}{1-\exp ({-}\tfrac{1}{2}\beta ^2_n-t^2)} \mathrm{d}t\,(1+o(1)), \end{aligned}$$

where

28 $$\begin{aligned} \beta _n^2 = s_n\left( \frac{1-\rho _n}{b_n+1}\right) ^2\left( 1+\frac{b_n}{\rho _n}\right) . \end{aligned}$$

Furthermore, the leading order behavior of ${\mathbb {P}}(Q_{n}=0)$ and $\mathrm{Var}\,Q_n$ is given by

$$\begin{aligned} \exp \left[ \frac{1}{\pi }\,\frac{b_n+\rho _n}{b_n+1}\,\int _0^\infty \frac{\beta _n/\sqrt{2}}{\tfrac{1}{2}\beta ^2_n+t^2}\,\mathrm{ln}\,\left( 1-\mathrm{e}^{{-}\tfrac{1}{2}\beta ^2_n-t^2}\right) \mathrm{d}t\right] , \end{aligned}$$

and

29 $$\begin{aligned} \frac{\beta _n^3/\sqrt{2}}{\pi }\left( \frac{b_n+\rho _n}{1-\rho _n}\right) ^2\left( \frac{b_n+1}{b_n+\rho _n}+1\right) \int _0^\infty \frac{t^2}{(\tfrac{1}{2} \beta _n+t^2)^2}\, \frac{\exp ({-}\tfrac{1}{2}\beta _n-t^2)}{1-\exp ({-}\tfrac{1}{2}\beta _n^2-t^2)}\mathrm{d}t,\nonumber \\ \end{aligned}$$

respectively.

The proof of Theorem 2 requires asymptotic evaluation of the Pollaczek-type integrals (20)–(22), for which we shall use a *nonstandard* saddle point method. The saddle point method in its standard form is typically suitable for large deviation regimes, for instance excess probabilities, and it cannot be applied to asymptotically characterize other stationary measures such as the mean or mass at zero. Indeed, in the presence of overdispersion, the saddle point converges to one (as $n\rightarrow \infty$), which is a singular point of the integrand, and renders the standard saddle point method useless. Our nonstandard saddle point method, originally proposed by [15] and also applied in [22], aims specifically to overcome this challenge. Subsequently, we apply the nonstandard saddle point method to turn these contour integrals into practical approximations. In contrast to the setting of [22], the analyticity radius tends to one in the setting with overdispersion, which is a singular point of the integrand. For the proof of Theorem 2, we therefore modify the special saddle point method developed in [22] to account for this circumstance.

### Proof

Our starting point is the probability generating function of the number of arrivals per time slot, given in (11), which is analytic for $|z|<1+1/b_n=:r_0$. Under Assumption 2, we consider ${\mathbb {E}}{Q}_n$ as given in (20). We set

30 $$\begin{aligned} g(z) = -\mathrm{ln }\,z+\frac{1}{s_n}\,\mathrm{ln }\big [{\tilde{A}}_{n}(z)\big ] = -\mathrm{ln }\,z - \frac{a_n}{s_n}\,\mathrm{ln }\left( 1+(1-z)b_n\right) , \end{aligned}$$

to be considered in the entire complex plane with branch cuts $(-\infty ,0]$ and $[r,\infty )$. The relevant saddle point $z_\mathrm{sp}$ is the unique zero *z* of $g'(z)$ with $z\in (1,r_0)$. Since

31 $$\begin{aligned} g'(z) = -\frac{1}{z} + \frac{\rho _n}{1+(1-z)b_n}, \end{aligned}$$

this yields

32 $$\begin{aligned} 1+(1-z_{\mathrm{sp}})b_n = \rho _n z_{\mathrm{sp}},\quad \mathrm{i.e., } \quad z_{\mathrm{sp}} = 1+\frac{1-\rho _n}{\rho _n+b_n}. \end{aligned}$$

We then find

33 $$\begin{aligned} {\mathbb {E}}{Q}_n = \frac{s_n}{2\pi i} \int _{|z| = 1+\varepsilon } \frac{g'(z)}{z-1}\,\frac{\exp (s_n\,g(z))}{1-\exp (s_n\,g(z))}\mathrm{d}z, \end{aligned}$$

and take $1+\varepsilon = z_{\mathrm{sp}}$. There are no problems with the branch cuts since we consider $\exp (s_ng(z))$ with integer $s_n$.

We continue as in [22] and thus we intend to substitute $z=z(v)$ in the integral in (33), where *z*(*v*) satisfies

$$\begin{aligned} g(z(v)) = g(z_{\mathrm{sp}})-\tfrac{1}{2}\,v^2\,g''(z_{\mathrm{sp}}) =: q(v) \end{aligned}$$

in the range ${-}\tfrac{1}{2}\delta _n \le v\le \tfrac{1}{2} \delta _n$ with $\delta _n \rightarrow 0$ as $n\rightarrow \infty$. Note that this range depends on *n*, whereas these bounds $\pm \tfrac{1}{2} \delta _n$ remained bounded away from zero in [22]. This severely complicates the present analysis. We consider the approximate representation

34 $$\begin{aligned} \frac{-s_n\,g''(z_{\mathrm{sp}})}{2\pi i}\int _{-\tfrac{1}{2} \delta _n}^{\tfrac{1}{2} \delta _n}\frac{v}{z(v)-1}\,\frac{\exp (s_n\,q(v))}{1-\exp (s_n\, q(v))} \mathrm{d}v \end{aligned}$$

of ${\mathbb {E}}{Q}_n$. We have to operate here with additional care, since both the analyticity radius $r_0=1+1/b_n$ and the saddle point $z_\mathrm{sp}$ outside the unit circle tend to 1 as $n\rightarrow \infty$. Specifically, proceeding under the assumptions that $(1-\rho _n)^2a_n$ is bounded while $a_n\rightarrow \infty$ and $b_n\ge 1$, see Assumption 2, we have from (32) that

35 $$\begin{aligned} z_{\mathrm{sp}}-1=\frac{1-\rho _n}{b_n+\rho _n} = \frac{1-\rho _n}{b_n} + O\left( \frac{1-\rho _n}{b^2_n}\right) , \end{aligned}$$

where the *O*-term is small compared to $(1-\rho _n)/b_n$ when $b_n\rightarrow \infty$. Next, we approximate $r_0$, using that $r_0>1$ satisfies

$$\begin{aligned} {-}\mathrm{ln}\, r_0 - \frac{\rho _n}{b_n}\, \mathrm{ln}\,(1+(1-r_0)b_n) = 0. \end{aligned}$$

Write $r_0 = 1+u/b_n$, so that we get the equation

$$\begin{aligned} 0&= {-}\mathrm{ln}\,\left( 1+\frac{u}{b_n}\right) - \frac{\rho _n}{b_n}\,\mathrm{ln }(1-u)\\&= {-}\frac{u}{b_n}\left( 1-\rho _n-\tfrac{1}{2}\left( \frac{1}{b_n}+\rho _n\right) u-\tfrac{1}{3}\left( \frac{-1}{b^2_n}+\rho _n\right) u^2+\cdots \right) , \end{aligned}$$

where we have used the Taylor expansion of $\mathrm{ln}(1+x)$ at $x=0$. Thus, we find

$$\begin{aligned} u=\frac{2(1-\rho _n)}{\rho _n+1/b_n}+O(u^2) = 2(1-\rho _n)+O((1-\rho _n)^2)+O\left( \frac{1-\rho _n}{b_n}\right) , \end{aligned}$$

and so,

$$\begin{aligned} r_0 = 1+2\,\frac{1-\rho _n}{b_n}+O\left( \frac{(1-\rho _n)^2}{b_n}\right) + O\left( \frac{1-\rho _n}{b^2_n}\right) . \end{aligned}$$

In (34) we choose $\delta _n$ so large that the integral has converged within exponentially small error using $\pm \,\delta _n$ as integration limits and, at the same time, so small that there is a convergent power series

36 $$\begin{aligned} z(v) = z_{\mathrm{sp}}+iv+ \sum _{k=2}^\infty c_k(iv)^k, \quad \text {for } |v| \le \tfrac{1}{2} \delta _n. \end{aligned}$$

To achieve these goals, we supplement the information on *g*(*z*), as given by (30)–(32), by

37 $$\begin{aligned} g''(z)=\frac{1}{z^2}+\frac{\rho _nb_n}{(1+(1-z)b_n)^2},\quad g''(1) = 1+\rho _nb_n,\quad g''(z_{\mathrm{sp}}) =\frac{1}{z_\mathrm{sp}^2}\left( 1+\frac{b_n}{\rho _n}\right) .\nonumber \\ \end{aligned}$$

Now,

$$\begin{aligned} \exp (s_n\,q(v)) = \exp (s_n\,g(z_\mathrm{sp}))\exp (-\tfrac{1}{2}\,s_n\,g''(z_{\mathrm{sp}})\,v^2), \end{aligned}$$

and

$$\begin{aligned} s_n\, g''(z_{\mathrm{sp}})v^2 = s_n\,b_nv^2(1+o(1)) = a_n(b_n\,v)^2(1+o(1)). \end{aligned}$$

Therefore, (34) approximates ${\mathbb {E}}{Q}_n$ with exponentially small error when we take $\tfrac{1}{2} \delta _n$ of the order $1/b_n$.

We next aim at showing that we have a power series for *z*(*v*) as in (36) that converges for $|v|\le \tfrac{1}{2}\delta _n$, with $\tfrac{1}{2}\delta _n$ of the order $1/b_n$.

### Lemma 2

Let

$$\begin{aligned} r_n:=\frac{1}{2\,b_n}-(z_{\mathrm{sp}} -1 ),\quad m_n:= \tfrac{2}{3}\rho _nr_n\sqrt{\frac{b_n+\rho _n^{-1}}{b_n+\rho _n}}, \end{aligned}$$

where we assume $r_n>0$. Then, (36) holds with real coefficients $c_k$ satisfying

38 $$\begin{aligned} |c_k|\le \frac{r_n}{m_n^k},\quad k=2,3,\ldots . \end{aligned}$$

### Proof

We let

39 $$\begin{aligned} G(z):=\frac{2(g(z)-g(z_{\mathrm{sp}}))}{g''(z_\mathrm{sp})(z-z_{\mathrm{sp}})^2}. \end{aligned}$$

Then $G(z_{\mathrm{sp}})=1$ and so we can write (4) as

40 $$\begin{aligned} F(z):=(z-z_{\mathrm{sp}})\sqrt{G(z)} = i v \end{aligned}$$

when $|z-z_{\mathrm{sp}}|$ is sufficiently small. Since $F(z_\mathrm{sp})=0$, $F'(z_{\mathrm{sp}})=1$, the Bürmann–Lagrange inversion theorem implies validity of a power series as in (36), with real $c_k$ since *G*(*z*) is positive and real for real *z* close to $z_{\mathrm{sp}}$. We therefore just need to estimate the convergence radius of this series from below.

To this end, we start by showing that

41 $$\begin{aligned} \mathrm{Re}[g''(z)] > \frac{4}{9}\,\rho _n^2\frac{b_n+\rho _n^{-1}}{b_n+\rho _n},\quad |z-z_{\mathrm{sp}}|\le r_n. \end{aligned}$$

For this, we consider the representation

42 $$\begin{aligned} G(z) = 2\int _{0}^1\int _0^1 \frac{g''(z_\mathrm{sp}+s\,t(z-z_{\mathrm{sp}}))}{g''(z_{\mathrm{sp}})} \,t\mathrm{d}s\mathrm{d}t. \end{aligned}$$

We have, for $\zeta \in {\mathbb {C}}$ and $|\zeta -1|\le 1/2b_{n}\le 1/2$, from (37) that

43 $$\begin{aligned} \mathrm{Re}[g''(\zeta )] = \mathrm{Re}(1/\zeta ^2) + \rho _nb_n\,\mathrm{Re}\left[ \left( \frac{1}{1+(1-\zeta )b_n}\right) ^2\right] \ge \tfrac{4}{9}(1+\rho _nb_n). \end{aligned}$$

To show the inequality in (43), it suffices to show that

44 $$\begin{aligned} \min _{|\xi -1|\le 1/2} \mathrm{Re}\left( \frac{1}{\xi ^2}\right) = \frac{4}{9}. \end{aligned}$$

The minimum in (44) is assumed at the boundary $|\xi -1|=1/2$, and for a boundary point $\xi$ we write

$$\begin{aligned} \xi = 1+\tfrac{1}{2}\cos \theta +\tfrac{1}{2} i \sin \theta , \quad 0\le \theta \le 2\pi , \end{aligned}$$

so that

$$\begin{aligned} \mathrm{Re}\left( \frac{1}{\xi ^2}\right) = \frac{1+\cos \theta +\tfrac{1}{4}\cos 2\theta }{\left( \tfrac{5}{4}+\cos \theta \right) ^2}. \end{aligned}$$

Now

$$\begin{aligned} \frac{\mathrm{d}}{\mathrm{d}\theta } \left[ \frac{1+\cos \theta +\tfrac{1}{4}\cos 2\theta }{\left( \tfrac{5}{4}+\cos \theta \right) ^2}\right] = \frac{\sin \theta \,(1-\cos \theta )}{4\left( \tfrac{5}{4}+\cos \theta \right) ^3} \end{aligned}$$

vanishes for $\theta =0,\pi ,2\pi$, where $\mathrm{Re}(1/\xi ^2)$ assumes the values 4 / 9, 4, 4/9, respectively. This shows (44).

We use (44) with $\xi = \zeta$ and $\xi =1+(1-\zeta )b_n$, with

45 $$\begin{aligned} \zeta = \zeta (s,t) = z_{\mathrm{sp}} + s\,t\,(z-z_\mathrm{sp}),\quad 0\le s,\, t\le 1, \end{aligned}$$

where we take $\zeta$ such that $|\zeta -1|\le 1/2b_n$. It is easy to see from $1<z_{\mathrm{sp}}<1+1/2b_n$ that $|\zeta -1|\le 1/2b_n$ holds when $|z-z_{\mathrm{sp}}|\le r_n=1/2b_n-(z_{\mathrm{sp}}-1)$. We have, furthermore, from (32) that $0<g''(z_{\mathrm{sp}})\le 1+b_n/\rho _n$. Using this, together with (43) where $\zeta$ is as in (45), yields

$$\begin{aligned} \mathrm{Re}[G(z)] \le \frac{4}{9}\,\frac{1+\rho _nb_n}{1+b_n/\rho _n}\,2\,\int _0^1\int _0^1 t\,\mathrm{d}s\,\mathrm{d}t = \tfrac{4}{9}\,\rho _n^2\,\frac{b_n+\rho _n^{-1}}{b_n+\rho _n} \end{aligned}$$

when $|z-z_{\mathrm{sp}}|\le r_n$, and this is (41). We therefore have from (40) that

$$\begin{aligned} |F(z)|>r_n\cdot \frac{2}{3}\rho _n\sqrt{\frac{b_n+\rho _n^{-1}}{b_n+\rho _n}} = m_n,\quad |z-z_{\mathrm{sp}}|=r_n. \end{aligned}$$

Hence, for any *v* with $|v|\le m_n$, there is exactly one solution $z=z(v)$ of the equation $F(z)-iv=0$ in $|z-z_{\mathrm{sp}}|\le r_n$ by Rouché’s theorem [2]. This *z*(*v*) is given by

$$\begin{aligned} z(v) = \frac{1}{2\pi i}\,\int _{|z-z_{\mathrm{sp}}|=r_n} \frac{F'(z)\,z}{F(z)-iv}\mathrm{d}z, \end{aligned}$$

and depends analytically on *v*, $|v|\le m_n$. From $|z(v)-z_\mathrm{sp}|\le r_n$, we can finally bound the power series coefficients $c_k$ according to

$$\begin{aligned} |c_k| = \left| \frac{1}{2\pi i}\int _{|iv|=m_n} \frac{z(v)-z_{\mathrm{sp}}}{(iv)^{k+1}}\mathrm{d}(iv)\right| \le \frac{r_n}{m_n^k}, \end{aligned}$$

and this completes the proof of the lemma. $\square$

### Remark 1

We have $z_{\mathrm{sp}}-1=o(1/b_n)$, see (35), and so

$$\begin{aligned} r_n = \frac{1}{2b_n}(1+o(1)),\quad m_n = \frac{1}{3b_n}(1+o(1)), \end{aligned}$$

implying that the radius of convergence of the series in (36) is indeed of order $1/b_n$ (since we have assumed $b_n\ge 1$).

We let $\delta _n=m_n$, and we write, for $0\le v\le \tfrac{1}{2}\delta _n$,

$$\begin{aligned} \frac{v}{z(v)-1}+\frac{{-}v}{z({-}v)-1} = \frac{-2iv\,\mathrm{Im}(z(v))}{|z(v)-1|^2}, \end{aligned}$$

where we have used that all $$c_k$$ are real, so that $$z(-v)=z(v)^*$$, where $$ ^*$$ denotes the complex conjugate. Now, from (38) and realness of the $$c_k$$, we have

46 $$\begin{aligned} \mathrm{Im}(z(v)) = v+\sum _{l=1}^\infty c_{2l+1}(-1)^l\,v^{2l+1} = v+O(v^3), \end{aligned}$$

and in similar fashion

47 $$\begin{aligned} |z(v)-1|^2 = (z_{\mathrm{sp}}-1)^2+v^2+O((z_{\mathrm{sp}}-1)^2v^2) + O(v^4) \end{aligned}$$

when $0\le v\le \tfrac{1}{2}\delta _n$. The order terms in (46), (47) are negligible in leading order, and so we get for $\mu _{Q_{n}}$ via (34) the leading order expression

$$\begin{aligned} \frac{{-}s_n\,g''(z_{\mathrm{sp}})}{2\pi i}\,\int _0^{\tfrac{1}{2}\delta _n}\frac{{-}2iv^2}{(z_\mathrm{sp}-1)^2+v^2}\,\frac{\exp (s_n\,q(v))}{1-\exp (s_n\, q(v))}\mathrm{d}v. \end{aligned}$$

We finally approximate $q(v) = g(z_{\mathrm{sp}})-\tfrac{1}{2} g''(z_\mathrm{sp})v^2$. There is a $z_1$, $1\le z_1\le z_{\mathrm{sp}}$, such that

$$\begin{aligned} g(z_{\mathrm{sp}}) = {-}\tfrac{1}{2}(z_{\mathrm{sp}}-1)^2\,g''(z_1), \end{aligned}$$

and, see (35) and (37),

$$\begin{aligned} g''(z_1) = g''(z_{\mathrm{sp}}) + O((1-\rho _n)b_n). \end{aligned}$$

Hence

48 $$\begin{aligned} s_n\,q(v)&= {-}\tfrac{1}{2} s_n\,g''(z_{\mathrm{sp}})\,[(z_{\mathrm{sp}}-1)^2+v^2]+O((1-\rho _n)b_ns_n(z_{\mathrm{sp}}-1)^2)\nonumber \\&= {-}\tfrac{1}{2} s_n\,g''(z_{\mathrm{sp}})[(z_\mathrm{sp}-1)^2+v^2]+O((1-\rho _n)^2a_n), \end{aligned}$$

where (35) has been used, and $a_nb_n = s_n(1+o(1))$. Therefore, the *O*-term in (48) tends to 0 by our assumption that $(1-\rho _n)^2a_n$ is bounded. Thus, we get for $\mu _{Q_{n}}$ in leading order

49 $$\begin{aligned} \frac{s_n g''(z_{\mathrm{sp}})}{\pi } \int _{0}^{\tfrac{1}{2}\delta _n}\frac{v^2}{(z_{\mathrm{sp}}-1)^2+v^2}\, \frac{\exp (-\tfrac{1}{2} g''(z_{\mathrm{sp}})s_n((z_\mathrm{sp}-1)^2+v^2))}{1-\exp (-\tfrac{1}{2} g''(z_{\mathrm{sp}})s_n((z_\mathrm{sp}-1)^2+v^2))} \mathrm{d}v.\nonumber \\ \end{aligned}$$

When we substitute $t=v\sqrt{s_n\,g''(z_{\mathrm{sp}})/2}$ and extend the integration in (49) to all $t\ge 0$ (at the expense of an exponentially small error), we get for $\mu _{Q_{n}}$ in leading order

$$\begin{aligned} =\frac{1}{\pi }\,\sqrt{2\,s_n\,g''(z_\mathrm{sp})}\,\int _{0}^\infty \frac{t^2}{\tfrac{1}{2}\beta _n^2}\,\frac{\exp ({-}\tfrac{1}{2}\beta ^2_n-t^2)}{1-\exp ({-}\tfrac{1}{2}\beta ^2_n-t^2)}\mathrm{d}t, \end{aligned}$$

where

$$\begin{aligned} \beta ^2_n = s_n\,g''(z_{\mathrm{sp}})(z_{\mathrm{sp}}-1)^2. \end{aligned}$$

Now using (32) and (37), we get the result of Theorem 2. A separate analysis of $\beta _n$ is provided in Sect. 5.1. $\square$

## Main insights and numerics

Through Theorem 2, we can write (27) as

$$\begin{aligned} {\mathbb {E}}{Q}_n \approx \tilde{\sigma }_n\,{\mathbb {E}}[ M_{\beta _n}] \end{aligned}$$

with

50 $$\begin{aligned} \tilde{\sigma }_n = \beta _n \left( \frac{b_n+\rho _n}{1-\rho _n}\right) . \end{aligned}$$

This robust approximation for ${\mathbb {E}}{Q}_n$ is suggestive of the following two properties that extend beyond the mean system behavior, and hold at the level of approximating the queue by $\sigma _n$ times the Gaussian random walk:

(i) At the process level, the space should be normalized with $\sigma _n$, as in (8). The approximation (27) suggests that it is better to normalize with $\tilde{\sigma }_n$. Although $\tilde{\sigma }_n / \sigma _n \rightarrow 1$ for $n\rightarrow \infty$, the ${\tilde{\sigma }}_n$ is expected to lead to sharper approximations for finite *n*.
(ii) Again at the process level, it seems better to replace the original hedge $\beta$ by the robust hedge $\beta _n$. This thus means that the original system for finite *n* is approximated by a Gaussian random walk with drift $-\beta _n$. Apart from this approximation being asymptotically correct for $n\rightarrow \infty$, it is also expected to approximate the behavior better for finite *n*.

### Convergence of the robust hedge

We next examine the accuracy of the heavy-traffic approximations for ${\mathbb {E}}{Q}_n$ and $\sigma ^2_Q$, following Corollary 3 and Theorem 2. We expect the robust approximation to be considerably better than the classical approximation when $\beta _n$ and $\tilde{\sigma }_n$ differ substantially from their limiting counterparts. Before substantiating this claim numerically, we present a result on the convergence rates of $\beta _n$ to $\beta$ and $\tilde{\sigma }_n$ to $\sigma _n$.

#### Proposition 1

Let $a_n,b_n$ and $s_n$ be as in Assumption 2. Then

51 $$\begin{aligned} \beta _n^2 = \beta ^2\left( 1 - \frac{1}{1+b_n+\sigma _n/\beta }\right) . \end{aligned}$$

#### Proof

From (28), we have

$$\begin{aligned} \beta _n^2&= s_n\left( \frac{1-\rho _n}{b_n+1}\right) ^2\left( 1+\frac{b_n}{\rho _n}\right) = \frac{1}{s_n}\left( \frac{s_n-a_nb_n}{b_n+1}\right) ^2\left( 1+\frac{s_n}{a_n}\right) \\&= \frac{1}{s_n}\frac{\beta ^2\,a_nb_n(b_n+1)}{(b_n+1)^2}\left( 1+\frac{s_n}{a_n}\right) = \beta ^2\,\frac{b_n}{b_n+1}\,\left( 1+\frac{a_n}{s_n}\right) =:\beta ^2\,{\bar{F}}_n. \end{aligned}$$

Now,

$$\begin{aligned} \bar{F_n}&= \frac{b_n}{b_n+1}\,\left( 1+\frac{a_n}{s_n}\right) = \frac{b_n}{b_n+1}+\frac{1}{b_n+1}\,\frac{a_nb_n}{s_n}\\&= 1-\frac{1}{b_n+1}\,\left( 1-\frac{a_nb_n}{s_n}\right) = 1-\frac{1}{b_n+1}\,\frac{\beta \,\sigma _n}{s_n}\\&= 1-\frac{1}{b_n+1}\,\frac{1}{1+\frac{\mu _n}{\beta \sigma _n}} = 1-\frac{1}{b_n+1+\frac{1}{\beta }\sqrt{a_nb_n(b_n+1)}}, \end{aligned}$$

which, together with $\sigma _n^2=a_nb_n(b_n+1)$, proves the proposition. $\square$

Note that $\beta _n$ always approaches $\beta$ from below. Also, (51) shows that $b_n$ is the dominant factor in determining the rate of convergence of $\beta _n$.

#### Proposition 2

Let $\tilde{\sigma }_n$ as in (50). Then

$$\begin{aligned} \tilde{\sigma }_n = \sigma _n + b_n\beta _n + O(1). \end{aligned}$$

#### Proof

Straightforward calculations give

$$\begin{aligned} \tilde{\sigma }_n&= \beta _n\,\left( \frac{s_nb_n+a_nb_n}{s_n-a_nb_n}\right) \\&= \frac{\beta _n}{\beta }\,\frac{b_n}{\sigma _n}\,(s_n+a_n) = \frac{\beta _n}{\beta }\,\sqrt{\frac{b_n}{a_n(b_n+1)}}\left( a_n(b_n+1)+\beta \sqrt{a_nb_n(b_n+1)}\right) \\&= \frac{\beta _n}{\beta }\left( \sqrt{a_nb_n(b_n+1)}+\beta b_n\right) = \frac{\beta _n}{\beta }\,\sigma _n + \beta _n b_n. \end{aligned}$$

Applying Proposition 1 together with the observation

$$\begin{aligned} \sigma _n \sqrt{1 - \frac{1}{1+b_n+\sigma _n/\beta }} = \sigma _n(1 + O(1/\sqrt{a_n}b_n)) = \sigma _n + O(1) \end{aligned}$$

yields the result. $\square$

**Fig. 1:**
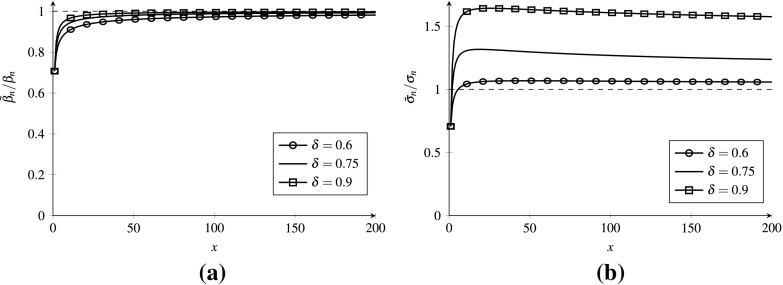
Convergence of the robust hedge. **a** Convergence of $\beta _n$, **b** convergence of $\tilde{\sigma }_n$

In Fig. 1, we visualize the convergence speed of both parameters in the case $\mu _n=n$, $\sigma _n = n^\delta$ with $\delta =0.7$ and $\beta =1$. This implies $a_n = n/(n^{2\delta }-1)$ and $b_n = n^{2\delta }-1$.

We observe that $\beta _n$ starts resembling $\beta$ fairly quickly, as predicted by Proposition 1; $\tilde{\sigma }_n$, on the other hand, converges extremely slowly to its limiting counterpart. Since ${\mathbb {E}}{Q}_n$ and $\mathrm{Var}\,Q_n$ are approximated by $\tilde{\beta }_n$ and $\tilde{\sigma }_n$, multiplied by a term that remains almost constant as *n* grows, the substitution of $\sigma _n$ by $\tilde{\sigma }_n$ is essential for obtaining accurate approximations, as we illustrate further in the next subsection.

**Table 1:** Numerical results for the Gamma–Poisson case with $\beta =1$ and $\delta =0.6$

$s_n$	$\rho _n$	${\mathbb {E}}Q_n$	(25)	(27)	$\sqrt{\mathrm{Var}\,Q_n}$	(26)	(29)
5	0.609	0.343	0.246	0.363	1.002	0.835	0.978
10	0.683	0.535	0.400	0.551	1.239	1.063	1.216
50	0.815	1.405	1.168	1.405	1.995	1.817	1.971
100	0.855	2.113	1.824	2.105	2.445	2.270	2.420
500	0.920	5.446	5.006	5.412	3.923	3.762	3.899

### Comparison between heavy-traffic approximations

We set $\mu _n=n$ and $\sigma ^2_n=n^{2\delta }$ with $\delta >\tfrac{1}{2}$, so that $s_n = n+\beta n^{\delta }$, and $a_n =n/(n^{2\delta -1}-1)$ and $b_n = n^{2\delta -1}-1$. Tables 1, 2, 3 and 4 present numerical results for various parameter values. The exact values are calculated using the method in Appendix B. Several conclusions are drawn from these tables. Observe that the heavy-traffic approximations based on the Gaussian random walk, (25) and (26), capture the right order of magnitude for both ${\mathbb {E}}Q_n$ and $\mathrm{Var}\,Q_n$. However, the values are off, in particular for small $s_n$ and relatively low $\rho _n := {\mathbb {E}}[A_{n}] / s_n$. The inaccuracy also increases with the level of overdispersion. In contrast, the approximations that follow from Theorem 2, (27) and (29), are remarkably accurate. Even for small systems with $s_n = 5$ or 10, the approximations for ${\mathbb {E}}Q_n$ are within 6$\%$ of the exact value for small $\rho _n$ and within $2\%$ for $\rho _n$ close to 1. For $\sigma _Q^2$, these percentages even reduce to $3\%$ and $1\%$, respectively. For larger values of $s_n$ these relative errors naturally reduce further. Overall, we observe that the approximations improve for heavily loaded systems, and the corrected approximations are particularly useful for systems with increased overdispersion.

**Table 2:** Numerical results for the Gamma–Poisson case with $\beta =1$ and $\delta =0.8$

$s_n$	$\rho _n$	${\mathbb {E}}Q_n$	(25)	(27)	$\sqrt{\mathrm{Var}\,Q_n}$	(26)	(29)
5	0.550	0.462	0.284	0.479	1.162	0.896	1.130
10	0.587	0.852	0.521	0.855	1.570	1.213	1.528
50	0.668	3.197	2.093	3.106	3.025	2.433	2.947
100	0.700	5.561	3.784	5.377	3.983	3.270	3.887
500	0.766	19.887	14.741	19.202	7.514	6.455	7.361

**Table 3:** Numerical results for the Gamma–Poisson case with $\beta =0.1$ and $\delta =0.6$

$s_n$	$\rho _n$	${\mathbb {E}}Q_n$	(25)	(27)	$\sqrt{\mathrm{Var}\,Q_n}$	(26)	(29)
5	0.949	11.532	11.306	11.495	3.634	3.559	3.602
10	0.961	17.565	17.268	17.548	4.474	4.398	4.444
50	0.979	46.368	45.869	46.418	7.241	7.168	7.218
100	0.984	70.340	69.735	70.430	8.910	8.839	8.888
500	0.991	184.900	183.989	185.108	14.422	14.357	14.404

**Table 4:** Numerical results for the Gamma–Poisson case with $\beta =0.1$ and $\delta =0.8$

$s_n$	$\rho _n$	${\mathbb {E}}Q_n$	(25)	(27)	$\sqrt{\mathrm{Var}\,Q_n}$	(26)	(29)
5	0.931	15.730	15.209	15.909	4.276	4.127	4.233
10	0.939	27.561	26.672	27.958	5.652	5.466	5.605
50	0.955	100.660	97.967	102.070	10.760	10.476	10.698
100	0.961	175.591	171.360	177.818	14.189	13.855	14.117
500	0.971	638.097	626.346	644.105	26.963	26.490	26.864

## A Proofs of convergence results

This section presents the details of the proofs of Lemma 1 and Theorem 1, using the random walk perspective of the process $\{Q_{k,n}\}_{k=0}^\infty$. This section is structured as follows. The next two lemmata are necessary for proving the first assertion of Theorem 1, concerning the weak convergence of the scaled process to the maximum of the Gaussian random walk, which is summarized in Proposition 4. The two remaining propositions of this section show convergence of ${\hat{Q}}_{n}$ at the process level as well as in terms of the three characteristics.

Let us first fix some notation:

52 $$\begin{aligned} Y_{k,n} := {\hat{A}}_{k,n}-\beta ,\quad S_{k,n} = \sum _{i=1}^k Y_{i,n}, \end{aligned}$$

with $S_{0,n} = 0$ and $k=1,2,\ldots$. Then (6) can be rewritten as

53 $$\begin{aligned} {\hat{Q}}_{n} {\;\buildrel {d}\over = \;}\max _{0\le k} \left\{ \sum _{i=1}^k Y_{i,n}\right\} =: M_{\beta ,n}. \end{aligned}$$

Last, we introduce the sequence of independent normal random variables $Z_1,Z_2,\ldots$ with mean ${-}\beta$ and unit variance 1, and

$$\begin{aligned} M_\beta {\;\buildrel {d}\over = \;}\max _{k\ge 0} \left\{ \sum _{i=1}^k Z_i\right\} . \end{aligned}$$

### A.1 Proof of Lemma 1

#### Proof

We show weak convergence of the random variable ${\hat{A}}_{n}$, as defined as the common distribution of (7), to a standard normal random variable. Since ${\hat{\Lambda }}_n$ is asymptotically standard normal, its characteristic function converges pointwise to the corresponding limiting characteristic function, i.e.,

54 $$\begin{aligned} \lim _{n\rightarrow \infty } \phi _{{\hat{\Lambda }}_n}(t) = \lim _{n\rightarrow \infty } \mathrm{e}^{-i\mu _nt/\sigma _n}\,\phi _{\Lambda _n}(t/\sigma _n) = \mathrm{e}^{{-}t^2/2},\quad \forall t\in {\mathbb {R}}. \end{aligned}$$

Furthermore, by the definition of $A_{n}$,

$$\begin{aligned} \phi _{A_{n}}(t) = {\mathbb {E}}\left[ \exp (\Lambda _n(\mathrm{e}^{it}-1))\right] = \phi _{\Lambda _n}\left( -i(\mathrm{e}^{it}-1)\right) , \end{aligned}$$

so that

55 $$\begin{aligned} \phi _{{\hat{A}}_{n}}(t) = \mathrm{e}^{-i\mu _nt/\sigma _n}\,\phi _{A_{k,n}}(t/\sigma _n) = \mathrm{e}^{-i\mu _nt/\sigma _n}\phi _{\Lambda _n}\left( -i(\mathrm{e}^{it/\sigma _n}-1)\right) . \end{aligned}$$

Now fix $t\in {\mathbb {R}}$. By using

$$\begin{aligned} -i(\mathrm{e}^{it/\sigma _n} - 1) = \frac{t}{\sigma _n} + O\left( t^2/\sigma _n^2\right) , \end{aligned}$$

we expand the last term in (55),

$$\begin{aligned} \phi _{\Lambda _n}(t/\sigma _n) + O\left( t^2/\sigma _n^2\right) \phi _{\Lambda _n}'(\zeta ) \end{aligned}$$

for some $\zeta$ such that $|\zeta - t/\sigma _n| < |i(1-\mathrm{e}^{it/\sigma _n})-t/\sigma _n|$. Also,

56 $$\begin{aligned} |\phi _{\Lambda _n}'(u)|&= \left| \frac{\delta }{\mathrm{d}u}\int _{-\infty }^\infty \mathrm{e}^{iux}\mathrm{d}F_{\Lambda _n}(x)\right| = \left| \int _{0}^{\infty } ix\,\mathrm{e}^{iux}\mathrm{d}F_{\Lambda _n}(x)\right| \nonumber \\&\le \int _{-\infty }^\infty |ix\,\mathrm{e}^{iux}|\,\mathrm{d}F_{\Lambda _n}(x) = \int _0^\infty x\mathrm{d}F_{\Lambda _n}(x) = \mu _n \end{aligned}$$

for all $u\in {\mathbb {R}}$. This implies

$$\begin{aligned} \phi _{{\hat{A}}_{k,n}}(t) = \phi _{\Lambda _n}(t/\sigma _n) + O\left( t^2\mu _n/\sigma _n^2\right) , \end{aligned}$$

in which the order term tends to zero as $n\rightarrow \infty$ by our assumption that $\mu _n/\sigma _n^2\rightarrow 0$. Combining this with (54), we find that $\phi _{{\hat{A}}_{n}}(t)$ converges to $\mathrm{e}^{{-}t^2/2}$ for all $t\in {\mathbb {R}}$, so that we can conclude by Lévy’s continuity theorem that ${\hat{A}}_{k,n} {\;\buildrel {d}\over \Rightarrow \;}N(0,1)$. $\square$

### A.2 Proof of Theorem 1

To secure convergence in distribution of ${\hat{Q}}_{n}$ to $M_\beta$, i.e., the maximum of a Gaussian random walk with negative drift, the following property of the sequence $\{Y_{k,n}\}_{n\in {\mathbb {N}}}$ needs to hold. Because the sequence $\{Y_{k,n}\}_{k\in {\mathbb {N}}}$ is i.i.d. for all *n*, we omit the index *k* in this result and its proof.

#### Lemma 3

Let $Y_{n}$ be defined as in (52) with $\mu _n,\sigma _n^2 < \infty$ for all $n\in {\mathbb {N}}$. Then, the sequence $\{(Y_{n})^+\}_{n\in {\mathbb {N}}}$ is uniformly integrable.

#### Proof

Note that the sequence $\{Y_n\}_{n\in {\mathbb {N}}}$ has constant finite mean and variance equal to 0 and 1, respectively, which implies it is bounded in $L^2$. Since $(Y^+_n)^2 \le Y_n^2$, the sequence $\{Y_n^+\}_{n\in {\mathbb {N}}}$ is bounded in $L^2$ as well. It readily follows from elementary probability theory that any sequence bounded in $L^2$ is uniformly integrable; see, for example, [7]. $\square$

By combining the properties proved in Lemmas 1 and 3 with Assumption 1, the next result follows directly by [3, Thm. X6.1].

#### Proposition 3

Let ${\hat{Q}}_{n}$ be as in (53). Then,

$$\begin{aligned} {\hat{Q}}_{n}{\;\buildrel {d}\over \Rightarrow \;}M_\beta ,\quad \mathrm{as}\ n\rightarrow \infty . \end{aligned}$$

Although Proposition 3 tells us that the properly scaled $Q_{n}$ converges to a non-degenerate limiting random variable, it does not cover the convergence of its mean, variance and the empty-queue probability. In order to secure convergence of these performance measures as well, we follow an approach similar [37], using Assumptions 1 and 3.

#### Proposition 4

Let ${\hat{Q}}_{n}$ be as in (53), $\mu _n,\sigma _n^2 \rightarrow \infty$ such that both $\sigma _n^2/\mu _n\rightarrow \infty$ and ${\mathbb {E}}[(\max \{{\hat{A}}_n,0\})^m]$ is bounded in *n* for $m=3,4$. Then

$$\begin{aligned} {\mathbb {P}}({\hat{Q}}_{n}= 0)&\rightarrow {\mathbb {P}}(M_\beta = 0),\\ {\mathbb {E}}[{\hat{Q}}_{n}]&\rightarrow {\mathbb {E}}[M_\beta ],\\ \mathrm{Var}\,{\hat{Q}}_{n}&\rightarrow \mathrm{Var}\,M_\beta , \end{aligned}$$

as $$n\rightarrow \infty $$.

#### Proof

First, we recall that ${\hat{Q}}_{n}{\;\buildrel {d}\over = \;}M_{\beta ,n}$ for all $n\in {\mathbb {N}}$, so that ${\mathbb {P}}({\hat{Q}}_{n} = 0) = {\mathbb {P}}(M_{\beta ,n}=0)$, ${\mathbb {E}}[{\hat{Q}}_{n}]={\mathbb {E}}[M_{\beta ,n}]$ and $\mathrm{Var}\,\,{\hat{Q}}_{n}=\mathrm{Var}\,\,M_{\beta ,n}$ as defined in (52). Our starting point is Spitzer’s identity, see [3, p. 230],

57 $$\begin{aligned} {\mathbb {E}}[\mathrm{e}^{it M_{\beta ,n}}] = \exp \left( \sum _{k=1}^\infty \frac{1}{k} ({\mathbb {E}}[\mathrm{e}^{itS_{k,n}^+}]-1)\right) , \end{aligned}$$

with $S_{k,n}$ as in (52), and $M_{\beta ,n}$ the all-time maximum of the associated random walk. Simple manipulations of (57) give

58 $$\begin{aligned} \mathrm{ln}\,{\mathbb {P}}(M_{\beta ,n} = 0)&= -\sum _{k=1}^\infty \frac{1}{k}\,{\mathbb {P}}(S_{k,n} > 0), \end{aligned}$$

59 $$\begin{aligned} {\mathbb {E}}[M_{\beta ,n}]&= \sum _{k=1}^\infty \frac{1}{k} {\mathbb {E}}[S^+_{k,n}] = \sum _{k=1}^\infty \frac{1}{k}\int _0^\infty {\mathbb {P}}(S_{k,n} > x) \mathrm{d}x, \end{aligned}$$

60 $$\begin{aligned} \mathrm{Var}\,M_{\beta ,n}&= \sum _{k=1}^\infty \frac{1}{k} {\mathbb {E}}[(S^{+}_{k,n})^2] =\sum _{k=1}^\infty \frac{1}{k}\int _0^\infty {\mathbb {P}}(S_{k,n} > \sqrt{x}) \mathrm{d}x. \end{aligned}$$

By Lemma 1, we know

$$\begin{aligned} {\mathbb {P}}(S_{k,n}> y) = {\mathbb {P}}\left( {\sum _{i=1}^k} Y_{i,n}> y \right) \rightarrow {\mathbb {P}}\left( \sum _{i=1}^k Z_i> y\right) , \end{aligned}$$

for $n\rightarrow \infty$, where the $Z_i$ are independent and identically normally distributed with mean $-\beta$ and variance 1. Because equivalent expressions to (58)–(60) apply to the limiting Gaussian random walk, it is sufficient to show that the sums converge uniformly in *n*, so that we can apply dominated convergence to prove the result.

We start with the empty-queue probability. To justify interchangeability of the infinite sum and limit, note

$$\begin{aligned} {\mathbb {P}}(S_{k,n}> 0) \le {\mathbb {P}}(|S_{k,n}+k\beta | > k\beta )\le \frac{k}{\beta ^2k^2} = \frac{1}{\beta ^2k}, \end{aligned}$$

where we used that ${\mathbb {E}}[ S_{k,n}] = k{\mathbb {E}}[Y_{1,n}] = -k\beta$ and $\mathrm{Var}\,S_{k,n} = k$, and apply Chebyshev’s inequality, so that

$$\begin{aligned} \sum _{k=1}^\infty \frac{1}{k}{\mathbb {P}}(S_{k,n} > 0) \le \sum _{k=1}^\infty \frac{1}{\beta ^2 k^2} < \infty , \quad \forall n\in {\mathbb {N}}. \end{aligned}$$

Hence,

$$\begin{aligned} \lim _{n\rightarrow \infty } \mathrm{ln}\,{\mathbb {P}}({\hat{Q}}_{n}= 0)&= \lim _{n\rightarrow \infty } - \sum _{k=1}^\infty \frac{1}{k}{\mathbb {P}}(S_{k,n}> 0) = -\sum _{k=1}^\infty \frac{1}{k} \lim _{n\rightarrow \infty }{\mathbb {P}}(S_{k,n}> 0)\\&= -\sum _{k=1}^\infty \frac{1}{k} {\mathbb {P}}\left( {\sum _{i=1}^k} Z_i > 0\right) = \mathrm{ln}\,{\mathbb {P}}(M_\beta = 0). \end{aligned}$$

Finding a suitable upper bound on $\frac{1}{k}\int _0^\infty {\mathbb {P}}({\hat{Q}}_{n}>x) \mathrm{d}x$ and $\frac{1}{k}\int _0^\infty {\mathbb {P}}({\hat{Q}}_{n}>\sqrt{x}) \mathrm{d}x$ requires a bit more work. We initially focus on the former, and the latter follows easily. The following inequality from [32] proves to be very useful:

61 $$\begin{aligned} {\mathbb {P}}({\bar{S}}_k>y) \le C_r\,\left( \frac{k\,\sigma ^2}{y^2}\right) ^r + k\,{\mathbb {P}}(X>y/r), \end{aligned}$$

where ${\bar{S}}_k$ is the sum of *k* i.i.d. random variables distributed as *X*, with ${\mathbb {E}}[X] = 0$ and $\mathrm{Var}\,\, X=\sigma ^2$, $y > 0$, $r>0$ and $C_r$ a constant only depending on *r*. We take $r=3$ for brevity in the remainder of the proof, although any $r>2$ will suffice. We have, from (61) with $X={\hat{A}}_n$, so that ${\mathbb {E}}[X]=0,\ \mathrm{Var}\,\, X = 1$, and $r=3$, $y=x+k\beta$,

62 $$\begin{aligned} {\mathbb {P}}(S_{k,n}> x)= & {} {\mathbb {P}}\left( \sum _{i=1}^k {\hat{A}}_{i,n}> x+k\beta \right) \nonumber \\&\le C_3 \left( \frac{k}{(x+k\beta )^2}\right) ^3 +\, k\,{\mathbb {P}}\left( {\hat{A}}_{1,n}>\frac{x+k\beta }{3}\right) . \end{aligned}$$

The quantity $(k/(x+k\beta )^2)^3$ is independent of *n*, and we have

63 $$\begin{aligned} \sum _{k=1}^\infty \frac{1}{k}\int _0^\infty \left( \frac{k}{(x+k\beta )^2}\right) ^3\, \mathrm{d}x&= \sum _{k=1}^\infty k^2 \int _0^\infty \frac{\mathrm{d}x}{(x+k\beta )^6} \nonumber \\&= \sum _{k=1}^\infty \frac{k^2}{5(k\beta )^5} = \frac{1}{5\beta ^5} \sum _{k=1}^\infty \frac{1}{k^3} < \infty . \end{aligned}$$

Next, by assumption, there is an $M_3>0$ such that

64 $$\begin{aligned} {\mathbb {E}}[(\max \{{\hat{A}}_{1,n},0\})^3] = \int _0^\infty t^3 \, \mathrm{d}P_{{\hat{A}}_{1,n}}(t) \le M_3 \end{aligned}$$

for all $n=1,2,\ldots$. It follows that for all $x>0$, $k=1,2,\ldots$, and all $n=1,2,\ldots$,

65 $$\begin{aligned} {\mathbb {P}}\left( {\hat{A}}_{1,n} > \frac{x+k\beta }{3}\right)&= \int _{\frac{x+k\beta }{3}}^\infty \mathrm{d}P_{{\hat{A}}_{1,n}}(t) \le \int _{\frac{x+k\beta }{3}}^\infty \frac{t^3}{\left( \frac{x+k\beta }{3}\right) ^3} \, \mathrm{d}P_{{\hat{A}}_{1,n}}(t) \nonumber \\&\le \frac{27}{(x+k\beta )^3} \, {\mathbb {E}}[(\max \{{\hat{A}}_{1,n},0\})^3]\le \frac{27M_3}{(x+k\beta )^3}. \end{aligned}$$

The quantity $1/(x+k\beta )^3$ is independent of *n*, and we have

66 $$\begin{aligned} \sum _{k=1}^\infty \int _0^{\infty } \frac{\mathrm{d}x}{(x+k\beta )^3} = \sum _{k=1}^\infty \frac{1}{2k^2\beta ^2} < \infty . \end{aligned}$$

Thus we see that for all $x>0$, $k=1,2,\ldots$, and all $n=1,2,\ldots$,

67 $$\begin{aligned} \frac{1}{k}\,{\mathbb {P}}(S_{k,n} > x ) \le \frac{C_3 k^2}{(x+k\beta )^6} + \frac{27M_3}{(x+k\beta )^3}, \end{aligned}$$

with

68 $$\begin{aligned} \sum _{k=1}^\infty \int _0^\infty \left( \frac{C_3 k^2}{(x+k\beta )^6} + \frac{27 M_3}{(x+k\beta )^3}\right) \, \mathrm{d}x < \infty . \end{aligned}$$

By Lebesgue’s theorem on dominated convergence, it then follows that

69 $$\begin{aligned} \lim _{n\rightarrow \infty } {\mathbb {E}}[{\hat{Q}}_n]&= \lim _{n\rightarrow \infty } \sum _{k=1}^\infty \frac{1}{k}\int _0^\infty {\mathbb {P}}(S_{k,n}>x)\, \mathrm{d}x \nonumber \\&= \sum _{k=1}^\infty \int _0^\infty {\mathbb {P}}\left( \sum _{i=1}^k Z_i> x\right) \, \mathrm{d}x = {\mathbb {E}}[M_\beta ]. \end{aligned}$$

In a similar fashion, by using (61) with $y = \sqrt{x}+k\beta$, we have

70 $$\begin{aligned} {\mathbb {P}}(S_{k,n}> \sqrt{x} ) \le C_3 \left( \frac{k}{(\sqrt{x}+k\beta )^2} \right) ^3 + k\,{\mathbb {P}}\left( {\hat{A}}_{1,n} > \frac{\sqrt{x}+k\beta }{3}\right) . \end{aligned}$$

Now we have

71 $$\begin{aligned} \sum _{k=1}^\infty \frac{1}{k}\int _0^\infty \left( \frac{k}{(\sqrt{x}+k\beta )^2} \right) ^3 \mathrm{d}x = \frac{1}{10\beta ^4} \sum _{k=1}^\infty \frac{1}{k^2} < \infty , \end{aligned}$$

where we have used that, by partial integration,

72 $$\begin{aligned} \int _0^\infty \frac{\mathrm{d}x}{(\sqrt{x}+k\beta )^6} = \int _0^\infty \frac{2u\, \mathrm{d}u}{(u+k\beta )^6} = \frac{1}{10(k\beta )^4}. \end{aligned}$$

Next, by assumption, there in an $M_4>0$ such that

73 $$\begin{aligned} {\mathbb {E}}[(\max \{{\hat{A}}_{1,n},0\})^4] = \int _0^\infty t^4 \, \mathrm{d}P_{{\hat{A}}_{1,n}}(t) \le M_4 \end{aligned}$$

for all $n=1,2,\ldots$. Then, as in (65), we get that for all $x>0$, $k=1,2,\ldots$, and all $n=1,2,\ldots$,

74 $$\begin{aligned} {\mathbb {P}}\left( {\hat{A}}_{1,n}> \frac{\sqrt{x}+k\beta }{3}\right) \le \frac{81 M_4}{(\sqrt{x}+k\beta )^4}, \end{aligned}$$

while, as in (66) and (72),

75 $$\begin{aligned} \sum _{k=1}^\infty \int _0^\infty \frac{\mathrm{d}x}{(\sqrt{x}+k\beta )^4} = \sum _{k=1}^\infty \frac{1}{3k^2\beta ^2} < \infty . \end{aligned}$$

Therefore, for all $x>0$, $k=1,2,\ldots$, and all $n=1,2,\ldots$,

76 $$\begin{aligned} \frac{1}{k}\,{\mathbb {P}}\left( S_{k,n}> \sqrt{x}\right) \le \frac{C_3k^2}{(\sqrt{x}+k\beta )^6} + \frac{81 M_4}{(\sqrt{x}+k\beta )^4}, \end{aligned}$$

with

77 $$\begin{aligned} \sum _{k=1}^\infty \int _0^\infty \left( \frac{C_3k^2}{(\sqrt{x}+k\beta )^6} + \frac{81 M_4}{(\sqrt{x}+k\beta )^4}\right) \, \mathrm{d}x < \infty . \end{aligned}$$

Hence, we conclude, as in (69), that $\lim _{n\rightarrow \infty } \mathrm{Var}\,{\hat{Q}}_n = \mathrm{Var}\,M_\beta$. This completes the proof. $\square$

We shall now indicate that the condition in Proposition 4 on boundedness of ${\mathbb {E}}[(\max \{{\hat{A}}_n,0\})^m]$ in *n* for $m=3,4$ is not overly restrictive by showing that it is satisfied by the Poisson mixture $A_n$ considered in Sect. 2. With $p_{n,k} = {\mathbb {P}}(A_n = k)$, we have that the pgf ${\tilde{A}}_n$ of *A* is given by

78 $$\begin{aligned} {\tilde{A}}_n(z) = \left( \frac{1}{1+b_n-b_n z} \right) ^{a_n} = \sum _{k=0}^\infty p_{n,k} z^k. \end{aligned}$$

For any $m=1,2,\ldots$, we have

79 $$\begin{aligned} {\mathbb {E}}[(\max \{{\hat{A}}_n,0\})^m] = {\mathbb {E}}\left[ \left( \max \left\{ \frac{A_n-\mu _n}{\sigma _n},0\right\} \right) ^m\right] = \frac{1}{\sigma _n^m} \sum _{k\ge \mu _n} (k-\mu _n)^m p_{n,k}.\nonumber \\ \end{aligned}$$

We shall conduct a somewhat heuristical saddle point analysis for the Cauchy integral representation

80 $$\begin{aligned} p_{k-1} = \frac{1}{2\pi i} \oint \frac{1}{z^k} \left( \frac{1}{1+b-bz}\right) ^a\mathrm{d}z, \quad k\ge \mu , \end{aligned}$$

with integration along a circle with center 0 and radius less than $(1+b)/b$. For convenience, we have temporarily omitted the index *n* in $p_{n,k-1}$, $a_n$, $b_n$ and $\mu _n$. The saddle point $z_{\mathrm{sp}}$ lies on the positive real axis between 0 and $(1+b)/b$ and is obtained by setting $f'(z)=0$, where

81 $$\begin{aligned} f(z) = {-}\, a\, \mathrm{ln}(1+b-b z) - k\,\mathrm{ln} \, z. \end{aligned}$$

When $k\ge \mu = ab$, it is found that

82 $$\begin{aligned} z_{\mathrm{sp}} = 1+\frac{k-ab}{(k+a)b}. \end{aligned}$$

We have, moreover,

83 $$\begin{aligned} f(z_{\mathrm{sp}})= & {} {-}a\,\mathrm{ln}\left( 1-\frac{k-ab}{k+a}\right) - k\,\mathrm{ln} \left( 1+\frac{k-ab}{(k+a)b} \right) \nonumber \\= & {} {-} \frac{(k-ab)^2}{(k+a)b} \left( \tfrac{1}{2} + O\left( \frac{k-ab}{k+a}\right) \right) \end{aligned}$$

and

84 $$\begin{aligned} f''(z_{\mathrm{sp}}) = \frac{1}{z_{\mathrm{sp}}^2}\,\frac{k^2}{a} + \frac{k}{z_{\mathrm{sp}}^2} = ab^2 \left( 1 + O\left( \frac{k-ab}{ab}\right) + O\left( \frac{1}{b}\right) \right) . \end{aligned}$$

Thus, we find the saddle point approximation

85 $$\begin{aligned} p_{k-1} \approx \frac{1}{2\pi } \sqrt{\frac{2\pi }{f''(z_{\mathrm{sp}})}}\, \exp \left( f(z_{\mathrm{sp}}) \right) \approx \frac{1}{\sqrt{2\pi ab^2}} \, \exp \left( {-}\frac{(k-ab)^2}{2ab^2}\right) , \end{aligned}$$

with *k* restricted to the range $0\le k-ab = O(ab)$. This range of *k* is sufficiently large for the series and the integral in (86) below to have converged. We now use this approximation in (79). Thus we have, using $\sigma _n^2 = a_nb_n(b_n+1) \approx a_nb_n^2$ and $\mu _n = a_nb_n$,

86 $$\begin{aligned} {\mathbb {E}}[(\max \{ {\hat{A}}_n,0\} )^m ]&= \frac{1}{\sigma _n^m}\, \sum _{k\ge \mu _n} (k-\mu _n)^m p_{n,k}\nonumber \\&\approx \frac{1}{\sqrt{2\pi }}\left( \frac{1}{a_nb_n^2}\right) ^{\frac{m+1}{2}} \sum _{k\ge \mu _n} (k-\mu _n)^m \exp \left( {-} \frac{(k-\mu _n)^2}{2a_nb_n^2}\right) \nonumber \\&\approx \frac{1}{\sqrt{2\pi }} \left( \frac{1}{a_nb_n^2}\right) ^{\frac{m+1}{2}} \int _0^\infty t^m \exp \left( {-}\frac{t^2}{2a_nb_n^2}\right) \, \mathrm{d}t \nonumber \\&= \frac{2^{\frac{m-1}{2}}}{\sqrt{2\pi }} \, \mathrm{\Gamma }\left( \frac{m+1}{2}\right) . \end{aligned}$$

The final member of (86) is independent of *n*, and this provides evidence that ${\mathbb {E}}[(\max \{{\hat{A}}_n,0\})^m]$ is bounded.

## B Numerical procedures

An alternative characterization of the stationary distribution is based on the analysis in [10] and considers a factorization in terms of (complex) roots:

$$\begin{aligned} Q_{n}(w) = \frac{(s_n-{\mathbb {E}}[A_{n}])(w-1)}{w^{s_n}-{\tilde{A}}_{n}(w)}\,\prod _{k=1}^{s_n-1} \frac{w-z^n_k}{1-z^n_k}, \end{aligned}$$

where $z_1^n,z_2^n\ldots ,z_{s_n-1}^n$ are the $s_n-1$ zeros of $z^{s_n}-{\tilde{A}}_{n}(z)$ in $|z|<1$, yielding

$$\begin{aligned} {\mathbb {E}}Q_n= & {} \frac{\sigma _n^2}{2(s_n-\mu _n)}-\frac{s_n-1+\mu _n}{2} + \sum _{k-1}^{s_n-1} \frac{1}{1-z^n_k}, \\ {\mathbb {P}}(Q_{n}=0)= & {} \frac{s_n-\mu _A}{{\tilde{A}}_{n}(0)}\prod _{k=1}^{s-1}\frac{z^n_k}{z^n_k-1}, \end{aligned}$$

which for our choice of ${\tilde{A}}_{n}(z)$ becomes

$$\begin{aligned} {\mathbb {E}}Q_n= & {} \frac{a_nb_n(b_n+1)}{2\beta \sqrt{a_n}b_n}-\frac{2a_nb_n+\beta \sqrt{a_nb_n(b_n+1)}-1}{2}+\sum _{k=1}^{s_n-1} \frac{1}{1-z^n_k}, \\ {\mathbb {P}}(Q_{n}=0)= & {} \beta \sqrt{a_nb_n(b_n+1)}(1+b_n)^{a_n}\prod _{k=1}^{s_n-1} \frac{z^n_k}{z^n_k-1}, \end{aligned}$$

where $z_1,\ldots ,z_{s_n-1}$ denote the zeros of $z^{s_n} - {\tilde{A}}_{n}(z)$ in $|z|<1$. These zeros exist under the assumption $s_n > a_nb_n$; see [2]. A robust numerical procedure to obtain these zeros is essential for a base of comparison. We discuss two methods that fit these requirements. The first follows directly from [20].

### Lemma 4

Define the iteration scheme

87 $$\begin{aligned} z_k^{n,l+1} = w^n_k [{\tilde{A}}_{n}(z_k^{n,l})]^{1/s_n}, \end{aligned}$$

with $w^n_k = \mathrm{e}^{2\pi ik/s_n}$ and $z_k^{n,0}=0$ for $k=0,1,\ldots ,s_{n-1}$. Then $z_k^{n,l} \rightarrow z_k^n$ for all $k=0,1,\ldots ,s_n-1$ for $l\rightarrow \infty$.

### Proof

The successive substitution scheme given in (87) is the fixed point iteration scheme described in [20], applied to the pgf of our arrival process. The authors show that, under the assumption of ${\tilde{A}}_{n}(z)$ being zero-free within $|z|\le 1$, the zeros can be approximated arbitrarily closely, given that the function $[{\tilde{A}}_{n}(z)]^{1/s_n}$ is a contraction for $|z|\le 1$, i.e.,

$$\begin{aligned} \left| \frac{\mathrm{d}}{\mathrm{d}z}[{\tilde{A}}_{n}(z)]^{1/s_n}\right| < 1. \end{aligned}$$

In our case,

88 $$\begin{aligned} \left| \frac{\mathrm{d}}{\mathrm{d}z}[{\tilde{A}}_{n}(z)]^{1/s_n}\right| = \left| \frac{\mathrm{d}}{\mathrm{d}z}\left( 1+(1-z)b_n\right) ^{-a_n/s_n}\right| = \frac{a_nb_n}{s_n}\left| 1+(1-z)b_n\right| ^{-a_n/s_n-1}, \end{aligned}$$

where $a_nb_n/s_n = \rho _n$ is close to, but less than, 1 and

$$\begin{aligned} |1+(1-z)b_n| \ge |1+b_n|-|z|b_n = 1+(1-|z|)b_n \ge 1, \end{aligned}$$

when $|z|\le 1$. Hence, the expression in (88) is less than 1 for all $|z|\le 1$. Evidently, ${\tilde{A}}_{n}(z)$ is also zero-free in $|z|\le 1$. Thus, [20, Lemma 3.8] shows that $z_k^{n,l}$ as in (87) converges to the desired roots $z^n_k$ for all *k* as *l* tends to infinity. $\square$

### Remark 2

The asymptotic convergence rate of the iteration in (87) equals $\frac{\mathrm{d}}{\mathrm{d}z}[{\tilde{A}}_{n}(z)]^{1/s_n}$ evaluated at $z=z_k^n$. Hence, convergence is slow for zeros near 1 and fast for zeros away from 1.

A different approach is based on the Bürmann–Lagrange inversion formula.

### Lemma 5

Let $w^n_k = \hbox {e}^{2\pi ik/s_n}$ and $\alpha _n = a_n/s_n$. Then, the zeros of $z^{s_n}-{\tilde{A}}_{n}(z)$ are given by

$$\begin{aligned} z_k^n = \sum _{l=1}^\infty \frac{1}{l!}\,\frac{\beta (l\alpha _n+l-1)}{\beta (l\alpha _n)}\,\frac{b_n+1}{b_n}\left( \frac{b_n}{(b_n+1)^{\alpha _n+1}}\right) ^l (w_k^n)^l, \end{aligned}$$

for $$k=0,1,\ldots ,s_n-1$$.

### Proof

Note that we are looking for *z*’s that solve

$$\begin{aligned} z\,[{\tilde{A}}_{n}(z)]^{-1/s_n} = z\left( 1+(1-z)b_n\right) ^{a_n/s_n} = w, \end{aligned}$$

where $w = w_k = \mathrm{e}^{2\pi i k/s_n}$. The Bürmann–Lagrange formula for $z=z(w)$, as can be found in [15, Sec. 2.2] for $z=z(w)$, is given by

$$\begin{aligned} z(w)&= \sum _{l=1}^\infty \frac{1}{l!}\,\left( \frac{\mathrm{d}}{\mathrm{d}z}\right) ^{l-1}\left[ \left( \frac{z}{z(1+(1-z)b_n)^{a_n/s_n}}\right) ^l\right] _{z=0}\,w^l\\&= \sum _{l=1}^\infty \frac{1}{l!}\,\left( \frac{\mathrm{d}}{\mathrm{d}z}\right) ^{l-1}\left[ \left( 1+(1-z)b_n\right) ^{-l\,a_n/s_n}\right] _{z=0}\,w^l. \end{aligned}$$

Set $\alpha _n = a_n/s_n$. We compute

$$\begin{aligned} \left( \frac{\mathrm{d}}{\mathrm{d}z}\right) ^{l-1}\left[ (1+(1-z)b_n)^{-l\alpha _n}\right] _{z=0} = \frac{\beta (l\alpha _n+l-1)}{\beta (l\alpha _n)}\,\frac{1+b_n}{b_n}\,\left( \frac{b_n}{(1+b_n)^{\alpha _n+1}}\right) ^l. \end{aligned}$$

With $c_n = b_n/(1+b_n)^{\alpha _n+1}$ and $d_n = (1+b_n)/b_n$, we thus have

$$\begin{aligned} z(w) = d_n\,\sum _{l=1}^\infty \frac{\beta (l\alpha _n+l-1)}{\beta (l+1)\beta (l\alpha _n)} c_n^l\,w^l. \end{aligned}$$

By Stirling’s formula

$$\begin{aligned} \frac{\beta (l\alpha _n+l-1)}{\beta (l+1)\beta (l\alpha _n)} = \frac{D}{l\sqrt{l}}\left( \frac{(\alpha _n+1)^{\alpha _n+1}}{\alpha _n^{\alpha _n}}\right) ^l, \end{aligned}$$

where $$D=\alpha _n^{1/2}(\alpha _n+1)^{-3/2}(2\pi )^{-1/2}$$. Now,

$$\begin{aligned} \frac{(\alpha _n+1)^{\alpha _n+1}}{\alpha _n^{\alpha _n}}\, c_n = \frac{(\alpha _n+1)^{\alpha _n+1}}{\alpha _n^{\alpha _n}}\cdot \frac{b_n}{(1+b_n)^{\alpha _n+1}} = \left( \frac{b_n+\rho _n}{b_n+1}\right) ^{\rho _n/b_n + 1}\left( \frac{1}{\rho _n}\right) ^{\rho _n/b_n}. \end{aligned}$$

This determines the radius of convergence $r_{\mathrm{BL}}$ of the above series for *z*(*w*):

89 $$\begin{aligned} \frac{1}{r_{\mathrm{BL}}} := \left( \frac{b_n+\rho _n}{b_n+1}\right) ^{\rho _n/b_n + 1}\left( \frac{1}{\rho _n}\right) ^{\rho _n/b_n}. \end{aligned}$$

The derivative with respect to $\rho _n$ of the quantity

90 $$\begin{aligned} \left( 1+\frac{\rho _n}{b_n}\right) \mathrm{ln }\left( \frac{b_n+\rho _n}{b_n+1}\right) +\frac{\rho _n}{b_n}\,\mathrm{ln}\left( \frac{1}{\rho _n}\right) \end{aligned}$$

is given by

$$\begin{aligned} \frac{1}{b_n}\mathrm{ln }\left( \frac{b_n+\rho _n}{b_n\rho _n+\rho _n}\right) > 0 \end{aligned}$$

for $0<\rho _n<1$ and $b_n>0$. Furthermore, the quantity in (90) vanishes at $\rho _n=1$ and is therefore negative for $0<\rho _n<1$ and $b_n>0$. $\square$

### Remark 3

The formula for the radius of convergence in (89) clearly shows the decremental effect of both having a large $b_n$ and/or having $\rho _n$ close to 1. The quantities $\beta (l\alpha +l-1)/(\beta (l+1)\beta (l\alpha ))$ in the power series for *z*(*w*) are not very convenient for recursive computation, although normally $\alpha = a_n/s_n$ is a rational number.
